# Microsphere-Based IgM and IgG Avidity Assays for Human Parvovirus B19, Human Cytomegalovirus, and Toxoplasma gondii

**DOI:** 10.1128/mSphere.00905-19

**Published:** 2020-03-18

**Authors:** Yilin Wang, Lea Hedman, Visa Nurmi, Inga Ziemele, Maria F. Perdomo, Maria Söderlund-Venermo, Klaus Hedman

**Affiliations:** a Department of Virology, University of Helsinki, Helsinki, Finland; b Helsinki University Hospital, HUSLAB, Helsinki, Finland; c Department of Pediatrics, Rīga Stradiņš University, Riga, Latvia; University of Missouri—Kansas City School of Medicine

**Keywords:** intrauterine infection, B19, HCMV, *T. gondii*, IgM, IgG avidity, suspension immunoassay, multiplex, infection time

## Abstract

Human parvovirus B19 (here B19), human cytomegalovirus (HCMV), and Toxoplasma gondii infections during pregnancy can lead to severe complications. While traditional diagnosis of infections is mostly confined to one pathogen at a time, a multiplex array is a feasible alternative to improve diagnostic management and cost-efficiency. In the present study, for these three pathogens, we developed microsphere-based suspension immunoassays (SIAs) in multiplex and monoplex formats for the detection of antimicrobial IgM antibodies as well as corresponding chaotrope-based IgG avidity SIAs. We determined the diagnostic performances of the SIAs versus in-house and commercial reference assays using a panel of 318 serum samples from well-characterized clinical cohorts. All the newly developed assays exhibited excellent performance compared to the corresponding high-quality reference methods. The positive and negative percent agreements of the IgM SIAs in comparison with reference methods were 95 to 100% and 98 to 100%, and those of the IgG avidity SIAs were 92 to 100% and 95 to 100%, respectively. Kappa efficiency values between the SIAs and the corresponding reference assays were 0.91 to 1. Furthermore, with another panel comprising 391 clinical samples from individuals with primary infection by B19, HCMV, or T. gondii, the IgM SIAs were highly sensitive for the detection of acute infections, and the IgG avidity SIAs were highly specific for the separation of primary infections from past immunity. Altogether, the strategy of IgM multiplex screening followed by IgG avidity reflex testing can provide high-throughput and accurate means for the detection and stage determination of B19, HCMV, and T. gondii infections.

**IMPORTANCE** Human parvovirus B19, human cytomegalovirus, and Toxoplasma gondii are ubiquitous pathogens. Their infections are often asymptomatic or mild in the general population yet may be transmitted from mother to fetus during pregnancy. Maternal infections by these pathogens can cause severe complications to the fetus or congenital abnormalities. As a rule, the risk of maternal transmission is critically related to the infection time; hence, it is important to determine when a pregnant woman has acquired the infection. In this study, we developed new diagnostic approaches for the timing of infections by three pathogens. All the new assays appeared to be highly sensitive and specific, providing powerful tools for medical diagnosis.

## Abstract (precis)

Human parvovirus B19, human cytomegalovirus, and Toxoplasma gondii are ubiquitous pathogens. Their infections are often asymptomatic or mild in the general population yet may be transmitted from mother to fetus during pregnancy. Maternal infections by these pathogens can cause severe complications to the fetus or congenital abnormalities. As a rule, the risk of maternal transmission is critically related to the infection time; hence, it is important to determine when a pregnant woman has acquired the infection. In this study, we developed new diagnostic approaches for the timing of infections by three pathogens. All the new assays appeared to be highly sensitive and specific, providing powerful tools for medical diagnosis.

## INTRODUCTION

Human parvovirus B19 (here B19), human cytomegalovirus (HCMV), and Toxoplasma gondii cause infections worldwide. Although these infections are usually asymptomatic in immunocompetent individuals, they can lead to severe complications during pregnancy. Maternal B19 infection can cause spontaneous abortion, fetal hydrops, and intrauterine death (1, 2), whereas HCMV and T. gondii can cause central nervous system damage in the fetus and can lead to long-term sequelae, including sensorineural hearing loss and chorioretinitis, respectively (3, 4). As a rule, with many microbes, acquired primary, as opposed to secondary, maternal infection carries the highest maternofetal transmission rate (4–6). With B19, fetal complications tend to occur by the second trimester (7), and with HCMV or T. gondii, transplacental transmission is more frequent during late gestation (8–10), whereas the incidence of severe congenital disease is higher in early pregnancy (11, 12).

Since the risk to the fetus is critically related to the infection time, it is essential to accurately determine whether a pregnant woman has acquired a primary infection during gestation or earlier. The classical approach is the detection of antimicrobial immunoglobulin G (IgG) and IgM antibodies. Specific IgM is a sensitive indicator of recent primary infection with all these microbes, yet the antibody can persist in the blood for a long time (13–15) and can also reappear in HCMV or T. gondii secondary infections. An approach for the dating of primary infection, e.g., with each of these pathogens, is the measurement of the antigen-binding avidity (functional affinity) of antimicrobial IgG. Upon initial antigenic challenge, IgG matures from low to high avidity due to antigen-driven B-cell selection and narrowing of the range of epitopes. This feature has been used in the clinical laboratory setting to distinguish between acute and past immunity via the implementation of either of two approaches: the use of a chaotropic agent to disrupt weak antigen-antibody interactions (16) or the addition of antigens to the solution to compete for the capture of high-avidity antibodies (17).

Current serodiagnosis is mostly confined to the detection of one pathogen at a time. A convenient approach for high-throughput antibody detection is a suspension immunoassay (SIA) employing flow cytometric analysis of fluorescent bead sets. In a previous study, we developed SIAs for the simultaneous detection and identification of IgGs against these three pathogens (18). In the present study, we employ this technology for the simultaneous determination of antimicrobial IgMs. We furthermore introduce corresponding chaotrope-based IgG avidity SIAs for the identification and timing of the corresponding primary infections.

## RESULTS

### Diagnostic performances of IgM and IgG avidity SIAs.

The diagnostic performances of the assays were evaluated using different sample types that have been stratified here into two panels (Table 1). Panel 1 includes archival serum samples analyzed by high-standard commercial or in-house reference assays (see Materials and Methods and Table S1 in the supplemental material). Panel 2 includes follow-up serum samples from patients presenting with a profile of primary or secondary infection by HCMV, T. gondii, or B19. The antimicrobial IgM SIAs were run in a multiplex format, and the IgG avidity SIAs were run in a singleplex format.

**TABLE 1:** Sample panels used in the study

Sample panel and pathogen studied	Study population (references)	No. of samples
Panel 1		
HCMV	Archival samples from HUSLAB	97
T. gondii	Archival samples from HUSLAB	94
B19	Archival samples from HUSLAB	40
B19	87 medical students	87

Panel 2		
HCMV	52 follow-up patients with HCMV primary or secondary infection (35, 41)	149
	39 patients with primary infection	108
	13 patients with secondary infection	41
T. gondii	22 follow-up pregnant women with T. gondii primary infection (19, 42)	116
	9 pregnant women with IgM and low avidity of IgG in their first sample	48
	13 pregnant women who were IgG seroconverters	68
B19	66 follow-up children and adults with B19 primary infection (18, 24, 25, 40)	126

10.1128/mSphere.00905-19.4 TABLE S1 Characteristics of all the reference immunoassays used in this study. Download Table S1, DOCX file, 0.02 MB. Copyright © 2020 Wang et al. 2020 Wang et al. This content is distributed under the terms of the Creative Commons Attribution 4.0 International license.

### (i) Panel 1.

*(a) HCMV*. Altogether, 97 samples were studied for IgM as well as for IgG avidity, and results were compared to those of Abbott’s Architect assays. The IgM SIA had an overall agreement of 97.9% (94/96 samples) (Table 2), and the IgG avidity SIA had an overall agreement of 95.9% (93/97 samples) (Table 3). Among the latter four serum samples, three showed low avidity in the SIA and high avidity in the Architect assay, while the remaining sample showed borderline avidity in the SIA and low avidity in the Architect assay. The third method (Vidas) showed low (two samples) or borderline (two samples) avidity for these samples.

**TABLE 2:** Assay performances of the IgM SIAs

Assay and result	No. of samples with result by the indicated comparator assay			% positive agreement (95% CI)	% negative agreement (95% CI)	Overall agreement (kappa value) (95% CI)
	Positive	Equivalent	Negative			
CMV IgM SIA	Architect CMV IgM					
Positive	42	1	1	97.7 (88.0–99.9)	98.1 (89.7–100)	0.96 (0.90–1)
Borderline	1	0	0			
Negative	1	0	51			

T. gondii IgM SIA	Vidas Toxo IgM					
Positive	46	1	0	100 (92–100)	100 (92–100)	1 (0.95–1)
Borderline	0	0	0			
Negative	0	0	47			

B19 IgM SIA	Biotrin B19 IgM					
Positive	37^*a*^	0	0	95 (83.1–99.4)	100 (95.9–100)	0.96 (0.91–1)
Borderline	1^*a*^	0	0			
Negative	2^*a*^	0	87^*b*^			

a Samples were tested with the Biotrin B19 IgM test.

b Samples were tested with an in-house B19 IgM test.

**TABLE 3:** Assay performances of the IgG avidity SIAs

Assay and result	No. of samples with result by the indicated comparator assay			% positive agreement (95% CI)	% negative agreement (95% CI)	Overall agreement (kappa value) (95% CI)
	Low	Equivalent	High			
CMV IgG avidity SIA	Architect CMV IgG avidity					
Low	37	0	3	100 (90.8–100)	94.9 (85.9–98.9)	0.91 (0.83–1)
Borderline	1	0	0			
High	0	0	56			

T. gondii IgG avidity SIA	Vidas Toxo IgG avidity					
Low	47	0	0	100 (92–100)	100 (92–100)	1.0 (0.95–1.0)
Borderline	0	0	0			
High	0	0	47			

B19 VP1 IgG avidity SIA	In-house B19 IgG VP2 ETS/in-house B19 VP1u IgG avidity EIA					
Low	36^*a*^	0	0^*b*^	92.3 (79.1–98.4)	100 (93.5–100)	0.93 (0.86–1.0)
Borderline	1^*a*^	0	2^*b*^			
High	2^*a*^	0	53^*b*^			

a Samples were tested with an in-house B19 IgG VP2 ETS test.

b Samples were tested with an in-house B19 VP1u IgG avidity EIA.

*(b)* T. gondii. IgM and IgG avidity were examined in 94 serum samples, and the results showed full concordance with the corresponding Vidas results (Tables 2 and 3).

*(c) B19.* IgM was tested in 40 clinical samples and 87 samples from students, and the results were compared to those of a reference IgM test (Table 2). The overall correspondence was 98.4% (125/127). All the student samples were B19 IgM negative by both the SIA and an enzyme immunoassay (EIA). Excluding three serum samples with insufficient VP1u IgG in the SIA, the concordance between the SIA and the reference IgG avidity assays was 96.8% (91/94) (Table 3).

### (ii) Panel 2.

**FIG 1:**
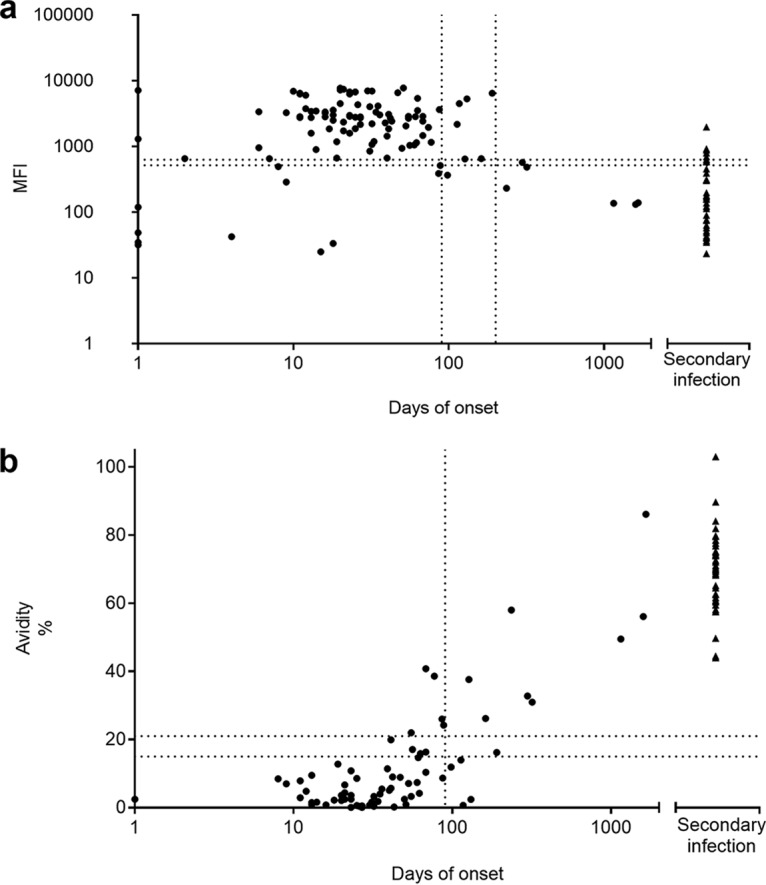
IgM response to (a) and avidity of IgG for (b) CMV in 39 subjects with CMV primary infection serologically monitored for up to 1,056 days (dots) and of 13 subjects with CMV secondary infection (triangles). The *y* axis shows MFI values (a) and avidity indices (b) by SIAs, and the *x* axis shows days after onset. The vertical dashed lines represent days 90 and 200 after the onset of symptoms. The horizontal dashed lines depict the IgM or IgG avidity cutoff values in SIAs.

*(a) HCMV.* In total, 108 samples from 39 patients with symptomatic primary infection were analyzed (Fig. 1). In the IgM SIA, positive results were found in 82.8% (48/58) of serum samples collected within 30 days of onset, in 94.6% (35/37) of serum samples collected during 30 to 90 days of onset, and in 58.8% (7/13) beyond 90 days of onset (Table 4). Over 200 days, all the sera were IgM SIA negative (Fig. 1a). The IgG avidity SIA showed low avidity in all 30 IgG-containing serum samples collected within 30 days of onset as well as in 74.2% (23/31) of the samples collected during 30 to 90 days. After 90 days, 53.8% (8/13) of the serum samples exhibited high avidity in the SIA. Beyond 200 days, all six serum samples showed high avidity (Fig. 1b). In addition, 41 samples from 13 patients with apparent reinfection/reactivation were studied with SIAs; 24.3% (10/41) were IgM SIA positive or borderline and overall showed lower IgM signals than in primary infections (Fig. 1a). All 41 samples exhibited a high avidity of IgG in the SIA, as they did in the reference test (Fig. 1b) (Table 4).

**TABLE 4:** IgM and IgG avidity SIA results with panel 2

Sampling	No. of samples with IgM SIA result			No. of samples with IgG avidity SIA result		
	Positive	Borderline	Negative	Low	Borderline	High
CMV						
<3 mo	83	0	12	53	3	5
>3 mo^*a*^	13	4	37	4	1	49

T. gondii^*b*^						
<3 mo	33	2	5	31	4	0
>200 days	27	2	21	9	6	35

B19						
<3 mo	82	0	0	68	4	1
>3 mo	11	1	32	0	2	42

a Samples collected from beyond 3 months of CMV primary and from CMV secondary infection.

b Samples collected from T. gondii study subgroups A and B.

**FIG 2:**
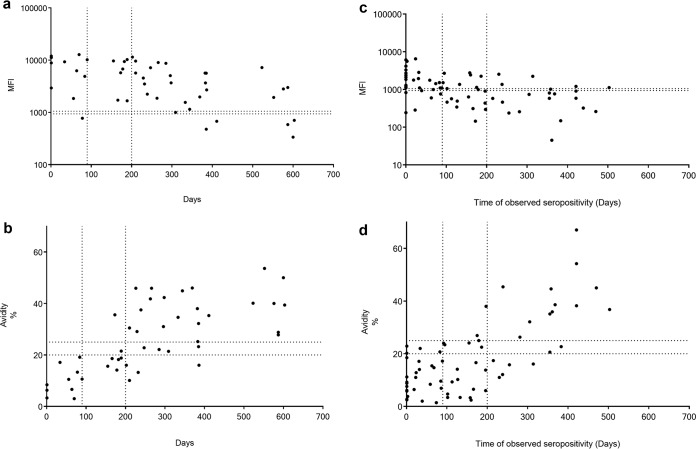
IgM response to (a and c) and avidity of IgG for (b and d) T. gondii. (a and b) IgM and IgG avidity values in subgroup A (9 patients who initially had low T. gondii avidity and were monitored for up to 603 days). (c and d) IgM and IgG avidity values in subgroup B (13 seroconverters monitored for up to 503 days after IgG seroconversion). The *y* axis shows MFI values (a and c) and avidity indices (b and d) by SIAs, while the *x* axis indicates days after the first IgM-positive sample (a and b) or days after IgG seroconversion (c and d). The vertical dashed lines represent days 90 and 200. The horizontal dashed lines depict the IgM or IgG avidity cutoff values in the SIAs.

*(b)* T. gondii. Subgroup A, comprising 48 samples from 9 patients with primary infection, was examined. In the SIA, a significant increase in IgG avidity was observed in 88.9% (8/9) of patients. All 11 samples collected within 90 days showed positive or borderline IgM SIA results (Fig. 2a) as well as low avidity by the SIA (Fig. 2b). Of the serum samples collected beyond 200 days, 80% (24/30) were still IgM SIA positive, and one was borderline (Fig. 2a), while the IgG avidities were high and borderline in 73% (22/30) and 13% (4/30) of the samples, respectively (Fig. 2b). Of the latter four low-avidity samples (>200 days), three came from a single patient (days 210, 232, and 386).

Moreover, in T. gondii subgroup B, constituting 68 samples from 13 seroconverters, examined with the SIA, 92.3% (12/13) of patients were IgM positive, and 76.9% (10/13) exhibited a significant IgG avidity increase at follow-up. Among the 29 samples collected within 90 days after T. gondii seroconversion, 82.8% (24/29) were IgM SIA positive or borderline (Fig. 2c), and all 24 samples showed low (*n* = 20) or borderline (*n* = 4) avidity by the SIA (Fig. 2d). Of the four samples with borderline avidity, two had been collected exceptionally late, 15 and 27 weeks after the IgG-negative serum samples were collected. Similar results were seen in the EIA (19). Of the 20 seropositive samples collected beyond 200 days, 75% (15/20) were IgM SIA negative (Fig. 2c), and 65% (13/20) showed high avidity and 10% (2/20) showed borderline avidity (Fig. 2d) in the SIA. All the samples collected beyond a year were of high avidity in the SIA, except for one that was borderline (avidity index, 21%).

**FIG 3:**
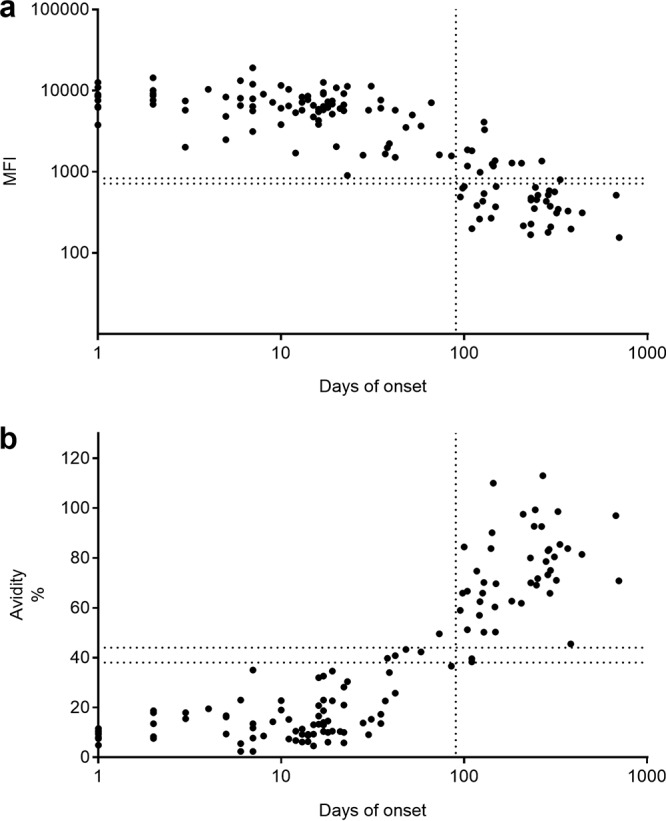
IgM response to B19 VP2 (a) and avidity of IgG for B19 VP1u (b). Represented are the IgM responses to B19 VP2 and IgG avidity for B19 VP1u in 80 subjects with symptomatic B19 infection serologically monitored for up to 700 days. The *y* axis shows MFI values (a) and avidity indices (b) by SIAs, and the *x* axis shows days after onset. The vertical dashed line represents day 90 after the onset of symptoms. The horizontal dashed lines depict the IgM or IgG avidity cutoff values in the SIAs.

*(c) B19.* We tested 126 serum samples from 66 children or adults with symptomatic B19 infection. In the SIA, B19 IgM was found in all 82 samples collected within 90 days of onset. Of the 44 samples obtained after day 90, 72.7% (32/44) were IgM SIA negative (Fig. 3a). All 61 serum samples collected within 30 days showed low avidity in the B19 SIA. Of the 12 samples collected at days 30 to 90, 58.3% (7/12) exhibited low avidity, 33% (4/12) exhibited borderline avidity, and 8.3% (1/12, on day 73) exhibited high avidity (Fig. 3b). Beyond 90 days, all samples had high or borderline avidity in the SIA (95.5% high and 4.5% borderline) (Fig. 3b and Table 4).

### Heterologous IgM reactivity.

Overall, heterologous IgM reactivities were observed in 3.5% (25/709) of the samples in this study. Of these, 8 samples belonged to panel 1 (6 HCMV IgM positive with low IgG avidity and 2 HCMV IgM negative with high IgG avidity), and 17 belonged to panel 2 (12 serum samples from 8 patients with HCMV primary infection, 3 serum samples from 2 patients with HCMV secondary infection, and 2 samples from a single patient with T. gondii primary infection). Among the 25 samples, 13 presented with homologous (cf. IgM) IgG. To identify the origin of the heterologous IgM reactivity, we performed multiplex IgG avidity SIAs and found that 11 samples showed high avidity against the homologous antigen, excluding recent primary infection by that pathogen (Tables 5 and 6). The other two samples were from a patient with a profile of HCMV secondary infection and displayed IgG and IgM SIA reactivities against B19 VP2 also. After retesting by the EIA, the VP2 IgM EIA was positive, and the VP2 IgG epitope-type-specificity (ETS) index was <10. Hence, it was apparent that this patient had a B19 primary infection inducing a serological pattern of HCMV secondary infection.

**TABLE 5:** Singleplex and multiplex avidity study of samples with heterologous IgM reactivities for HCMV, B19, and T. gondii

Study cohort	Sample type (day of onset)	Avidity index (%)			
		Singleplex CMV assay	Multiplex assay		
			CMV	B19	T. gondii
Panel 1	CMV IgM positive, low avidity of IgG	0.1	1.6		73.9
Panel 1	CMV IgM positive, low avidity of IgG	1.2	3	77.3	77.1
Panel 1	CMV IgM positive, low avidity of IgG	1.2	2	59	
Panel 1	CMV IgM negative, high avidity of IgG	65.7	64.7		84.4
Panel 2	CMV primary infection (16)	1.9	2.5		34.2
Panel 2	CMV primary infection (68)	10.4	10.9		49.6
Panel 2	CMV primary infection (18)	2.2	2.8		77.4
Panel 2	CMV Primary infection (34)	1.8	4.7		84.4
Panel 2	CMV Primary infection (19)	12.8	9		157.0

**TABLE 6:** Singleplex and multiplex avidity study of samples with heterologous IgM reactivities for T. gondii and B19

Study cohort	Sample type (days after first IgM-positive sample)	Avidity index (%)		
		Singleplex T. gondii assay	Multiplex assay	
			T. gondii	B19
Panel 2	T. gondii primary infection (0)	18	13.8	84.5
Panel 2	T. gondii primary infection (64)	6.5	6.6	76.9

### Reproducibilities of SIAs.

The intra- and interassay coefficients of variation (CVs) of HCMV, T. gondii, and B19 IgM SIAs were assessed using serum pools containing or lacking the specific IgMs. The respective intra-assay CVs were found to be 2 to 9%, 9 to 11%, and 6 to 9%, while the respective interassay CVs were 9 to 12%, 11 to 16%, and 11 to 14%. The respective interassay CVs of IgG avidity SIAs, assessed using acute-phase and past-infection serum pools, were 9 to 18%, 14 to 19%, and 14 to 17%, respectively.

## DISCUSSION

Combinations of serological tests are practicable for the detection of infections during pregnancy (20–23). While IgM in general is a sensitive indicator of recent primary infection, in many contexts, it lacks clinical specificity, hence calling for additional markers for infection dating. In such combinatory or “reflex” diagnostics, a feasible strategy is initial screening for antimicrobial IgG and IgM, in a multiplex format, followed by retesting of the IgM-positive samples with a conceptually different test, in singleplex, to attest the infection status. We have previously established microsphere-based IgG assays for B19, HCMV, and T. gondii infections (18). In this study, for these important pathogens, we successfully developed (i) IgM SIAs, for use as a primary approach (including screening), as well as (ii) the corresponding IgG avidity SIAs, for assessment of IgM-positive samples.

The new assays were validated here with 318 archival serum samples. Compared to high-quality commercial or in-house assays, the respective positive and negative percent agreements of the IgM SIAs were 95% to 100% and 98% to 100%, and those of the IgG avidity SIAs were 92% to 100% and 95% to 100%. Excellent agreement was seen between SIAs and reference assays for IgM (kappa coefficient 95% confidence interval [CI], 0.96 to 1) and for IgG avidity (kappa coefficient 95% CI, 0.91 to 1). In clarifying the B19 infection time, the new VP1u IgG avidity SIA also agreed well with the established EIA for the “conformation dependence” or “epitope type specificity” (ETS) of VP2 IgG, divergent qualitative determinants of antimicrobial IgG maturing progressively during the months since the first antigenic challenge (21, 24, 25).

Of note, the three HCMV IgG avidity tests used in the current study (SIA, Architect, and Vidas) are technologically distinct: (i) while the SIA and Vidas are based on protein denaturation, Architect utilizes antigen competition (17), and (ii) while Vidas and Architect are based on single dilutions of serum (urea treated/reference), the SIA is based on the endpoint titration of serum (dilution series). Notwithstanding the assay type differences, in diagnostic performance, the HCMV SIA agreed well with the corresponding Architect assay.

The IgM as well as the IgG avidity SIAs showed high clinical sensitivities among the samples collected within 3 months of primary infection by HCMV, T. gondii, or B19. Indeed, in HCMV and B19 IgG avidity SIAs, only beyond 2 months of symptom onset did the first instances of high avidity appear. As both persisting and reappearing IgMs were observed among these samples, the need for infection time verification became substantiated. With the IgG avidity SIAs, more than 90% of samples collected beyond 3 months of primary infection by B19 or HCMV (including secondary responses of the latter) were correctly identified (high avidity) as past infection. Likewise, the T. gondii IgG avidity SIA could effectively distinguish acute from latent/chronic T. gondii infections; however, low-avidity IgG was seen in five patients beyond 200 days of primary infection. Persistence of low-avidity IgG after T. gondii infection has been seen in many studies, especially among pregnant women and in medicated patients (26–29). Therefore, as pointed out previously (30), measurement of T. gondii IgG avidity serves better in ruling out than ruling in a recently acquired infection. NB, even if the newly developed assays in this study showed good reproducibility, the use of a calibrator serum could further increase their precision, particularly for low-positive and borderline results.

The antigens used for T. gondii IgM detection are usually tachyzoite lysates or recombinant proteins (17, 19, 31). Here, we employed a tachyzoite lysate enriched in membrane fractions, including the apical complex. The latter is associated with active motility during parasite invasion and is a strong immunogen for IgM (32). Interestingly, the presently generated IgM SIA based on this antigen showed 100% agreement with the Vidas IgM test employing the tachyzoite lysate.

IgM antibodies appear in circulation not only after primary or secondary infection but also as a result of polyclonal B-cell stimulation (33) with, e.g., transient heterologous IgM reactivity induced by HCMV primary infection, as has been known for a long time (34). Hence, the correct identification of the origin calls for another marker, such as a qualitative characteristic of the antimicrobial IgG (35). Also, to this end, the presently employed multiplex IgG avidity SIAs were shown to be suitable. In addition to field diagnosis, the utility of IgG avidity multiplexing has been noted in vaccine development (36).

Endpoint titration of serially diluted sera was successfully employed here in microsphere-based IgG avidity measurements. While such a procedure is unaffected by the IgG level in the sample, it calls for series of stepwise dilutions of the specimen. In this regard, a simpler approach based on a single dilution (37) could be an interesting choice for microsphere-based IgG avidity measurements.

Altogether, the IgM and IgG avidity SIAs were closely comparable to high-quality reference assays in diagnostic performance, providing reliable and cost-effective means for the diagnosis of B19, HCMV, and T. gondii infections. The new IgM assays were highly sensitive in the detection of recent primary infections, as were the new IgG avidity assays, which furthermore efficiently separated acute/primary infections from distant/secondary infections. The strategy of IgG-IgM multiplex screening followed by IgG avidity reflex testing provides a high-throughput, accurate means for the detection and stage determination of B19, HCMV, and T. gondii infections.

## MATERIALS AND METHODS

### Study samples and patients. (i) Panel 1.

Panel 1 included 231 archival (−20°C) serum samples sent to the Helsinki University Central Hospital Laboratory Service (HUSLAB) for diagnostic evaluation between 2003 and 2013 as well as 87 samples collected from constitutionally healthy medical students (Table 1). The main characteristics of the reference assays, including cutoffs, are summarized in Table S1 in the supplemental material.

*(a) HCMV*. Ninety-seven serum samples were examined for HCMV IgM antibodies and IgG antibody avidity using the respective Architect assays as reference tests (Abbott). In the Architect HCMV IgM assay, 44 serum samples were positive, 1 was borderline, and 52 were negative. In the Architect HCMV IgG avidity assay, 38 serum samples displayed low avidity, and 59 displayed high avidity. IgG avidity discordances between the SIA and Architect were assessed with the Vidas assay (bioMérieux).

*(b)* T. gondii. Ninety-four serum samples were analyzed for T. gondii IgM antibodies and IgG avidity using the Vidas Toxo IgM and IgG avidity assays as reference tests (bioMérieux). In the Vidas Toxo IgM assay, 46 samples were positive, 47 were negative, and 1 was borderline. In the Vidas Toxo IgG avidity assay, 47 serum samples were of low avidity, and 47 were of high avidity.

*(c) B19*. Forty serum samples were tested for B19 IgM antibodies by Biotrin’s B19 IgM assay (Liaison; DiaSorin) as well as for VP2 IgG epitope type specificity (ETS) by an in-house ETS EIA (38). All 40 serum samples showed the presence of B19 VP2 IgM antibodies and a low index of IgG ETS, indicating acute infection. On the other hand, among the 87 medical students, 57 were seropositive for B19 VP2 IgG yet devoid of VP2 IgM by the corresponding in-house EIAs (39). These 57 samples were studied for B19 IgG avidity by a VP1u antigen-based EIA (21, 25, 40, 41); 56 exhibited high avidity, indicating past B19 infection, and 1 was borderline.

### (ii) Panel 2.

Panel 2 included 391 serum samples from 140 patients with primary or secondary infections by HCMV, T. gondii, or B19. These patients and specimens have previously been examined serologically for HCMV (35, 41), T. gondii (19, 42), or B19 (18, 24, 25, 40).

*(a) HCMV.* A total of 108 samples originated from 39 patients with HCMV primary infection (35, 41) monitored serologically for up to 1,653 days. Among the samples, 58 had been collected within 30 days, 37 were collected within 30 to 90 days, and 13 were collected beyond 90 days from the onset of symptoms. These 39 patients with HCMV primary infection were apparently immunocompetent, except for a single heart transplant recipient. Moreover, 41 samples originated from 13 patients with a serological profile of HCMV secondary infection (exogenous reinfection or endogenous reactivation) (41). Of these 13 patients, 9 were transplant recipients (2 heart, 2 liver, 1 lung, 2 kidney, and 2 bone marrow recipients). The serum samples had been collected between 1986 and 1997, and the number of samples per patient ranged from 1 to 6.

*(b)* T. gondii. A total of 116 samples were obtained from 22 pregnant women with T. gondii primary infection (29), of whom 9 individuals presented with specific IgM and low-avidity IgG in their first samples, constituting subgroup A (*n* = 48 samples). These patients had been monitored serologically for a year (or more), except for one, who was monitored for 64 days. The other 13 patients were IgG seroconverters monitored for up to 503 days, constituting subgroup B (*n* = 68 samples). These serum samples had been collected between 1989 and 1990 (19, 42). The number of serum samples per patient ranged from 2 to 7.

*(c) B19.* A total of 126 serum samples were obtained from 66 children or adults (median age, 33 years; range, 2 to 55 years) with symptomatic B19 infection. The patients had been monitored serologically for up to 700 days. Collected between 1992 and 2001, there were 1 to 4 serum samples per patient (18, 24, 25, 40). Among the samples, 69 had been taken within 30 days, 13 samples were taken within 30 to 90 days, and 44 samples were taken beyond 90 days of onset.

### (iii) Diagnostic criteria of infections (panel 2).

*(a) HCMV.* The 39 patients with HCMV primary infection presented with seroconversion of HCMV IgG and a low avidity of HCMV IgG in the first positive sample, and 7 patients also had HCMV IgM seroconversion, whereas 32 patients were IgM positive or borderline with the first sample. The 13 patients with a profile of HCMV secondary infection had a 4-fold (or higher) increase of the HCMV IgG level, from the existing presence in the first sample of high-avidity HCMV IgG. Nine of these patients showed IgM seroconversion, 2 were IgM borderline, and 2 remained IgM negative.

*(b)* T. gondii. The 9 patients of T. gondii subgroup A exhibited T. gondii IgM as well as low-avidity IgG in the first sample, and the 13 patients in subgroup B showed seroconversion of T. gondii IgG. The seroconverters showed IgM seroconversion (*n* = 3) or were IgM positive or borderline with the first sample (*n* = 9), while a single patient lacked IgM. All IgG seroconverters were of low or borderline avidity with the first seropositive sample.

*(c) B19.* The 66 patients with recent B19 infection had B19 IgM (*n* = 66) as well as seroconversion (*n* = 25) or a significant rise (*n* = 41) in the level of B19 IgG and had low (<15%) avidity or a low (<10) ETS EIA ratio in the first seropositive sample.

### (iv) Ethics approval.

The Helsinki University Hospital Ethics Committee accepted the use of clinical samples in this study (Dnro 553/E6/2001, §106, 11.06.2014). The serum samples from medical students were obtained with informed consent. All other samples in this study were taken as part of standard care and were analyzed anonymously.

### Microsphere-based suspension immunoassays. (i) Coupling of antigens to magnetic microspheres.

The coupling of antigens to carboxylated fluorescent microspheres (Luminex Corp., USA) was performed according to the manufacturer’s protocol and as described previously by Wang et al. (18). The coupled microspheres were stored in StabilGuard (SG) buffer (SurModics, USA) at 4°C in the dark. Optimal antigen concentrations were determined by titration (ranging from 200 to 0.8 μg per 10^6^ microspheres). The conditions for each assay are presented in Tables 7 and 8.

**TABLE 7:** Antigens and suspension immunoassay conditions for IgM assays

Assay	Antigen	Concn (μg)/million microspheres	Source	Cutoff determination		
				Cutoff criterion	Reference value MFI	No. of seronegative samples
CMV IgM	Viral lysate (strain AD 169)	25	Advanced Biotechnologies	2 SD	Negative, ≤518	60
				3 SD	Positive, >631	

T. gondii IgM	T. gondii (RH strain) lysate enriched in membrane fraction, IgM grade	6	Microbix Biosystems	4 SD	Negative, ≤938	60
				5 SD	Positive, >1,056	

B19 VP2 IgM	In-house insect cell recombinant VP2	6	In-house	4 SD	Negative, ≤714	86
				5 SD	Positive, >831	

**TABLE 8:** Antigens and suspension immunoassay conditions for IgG avidity assays

Assay	Antigen	Concn (μg)/million microspheres	Source	Cutoff determination			
				Cutoff criterion	Reference value index	Primary infection	
						Time	No. of samples
CMV IgG avidity	Viral lysate (strain AD 169)	20	Advanced Biotechnologies	2.5 SD	Acute, ≤15	≤50 days	45
				4 SD	Past, >21		

T. gondii IgG avidity	T. gondii (RH strain) tachyzoite lysate	12.5	Microbix Biosystems	3 SD	Acute, ≤20	<3 mo	34
				4 SD	Past, >25		

B19 VP1u IgG avidity	Prokaryotic recombinant fusion protein containing the B19 VP1 unique region	50	In-house	3.5 SD	Acute, ≤38	≤28 days	60
				4.5 SD	Past, >44		

### (ii) Internal controls.

*(a) Naked microspheres*. For specificity, each test run included control uncoupled (“naked”) microspheres stored in SG buffer (18).

*(b) Rheumatoid factor (RF) control*. In the IgM test, each sample was also tested with human IgG (Sigma-Aldrich, USA)-coated microspheres to monitor the effectiveness of IgG removal (illustrated in Fig. S1). The coupling and storage of IgG-coated microspheres were the same as those for the antigen-coated magnetic microspheres (see above).

10.1128/mSphere.00905-19.2 FIG S1 Rheumatoid factor (RF) control. In the IgM SIA, each sample was tested with human IgG (Sigma-Aldrich, USA)-coupled microspheres. RF in serum can be semiquantified when RF bridges between human IgG-coated microspheres and biotinylated anti-human IgM. Download FIG S1, DOCX file, 0.4 MB. Copyright © 2020 Wang et al. 2020 Wang et al. This content is distributed under the terms of the Creative Commons Attribution 4.0 International license.

### (iii) Multiplex IgM SIA.

The multiplex IgM SIA included the removal of IgG with GullSORB (goat anti-human IgG; Meridian Bioscience, USA). According to our previous determination, described in Text S1 in the supplemental material, this pretreatment increased not only the specificity but also the sensitivity of the IgM assays (Fig. S2). The conditions for each IgM assay are presented in Table 7. In brief, GullSORB was mixed with serum, at a serum dilution of 1:20 (43). The mixture was kept at room temperature for 1 h with shaking and then centrifuged at 14,000 × *g* for 1 min to remove IgG precipitates. The supernatant was further diluted 4-fold. Next, 50 μl of this (1:80) IgG-depleted serum was incubated with 1.75 × 10^3^ antigen-coated (or control) microspheres/analyte/well for 45 min. After washes, 50 μl of biotinylated anti-human IgM (Sigma, USA) at 3 μg/ml was added for 30 min. After washes, 50 μl of 6 μg/ml streptavidin-conjugated phycoerythrin (SA-PE; Life Technologies, USA) in phosphate-buffered saline (PBS) with 0.05% Tween 20 (PBST) was applied for 20 min. After final washes, each well was resuspended in 120 μl of PBST and read on a Bio-Plex 200 instrument (Bio-Rad). The median fluorescence intensity (MFI) values were determined.

10.1128/mSphere.00905-19.1 TEXT S1 IgG depletion in IgM SIAs. Download Text S1, DOCX file, 0.01 MB. Copyright © 2020 Wang et al. 2020 Wang et al. This content is distributed under the terms of the Creative Commons Attribution 4.0 International license.

10.1128/mSphere.00905-19.3 FIG S2 IgG depletion in IgM SIAs. The pools containing or lacking specific HCMV IgM were used to compare the IgM SIA results before (blue) and after (red) IgG depletion, respectively. The *y* axis shows MFI values, and the *x* axis shows series of serum dilutions. The IgM response from each serum dilution is indicated: blue squares, IgM-positive pool without IgG depletion; red upward triangles, IgM-positive pool with IgG depletion; blue downward triangles, IgM-negative pool without IgG depletion; red diamonds, IgM-negative pool with IgG depletion. Download FIG S2, DOCX file, 0.1 MB. Copyright © 2020 Wang et al. 2020 Wang et al. This content is distributed under the terms of the Creative Commons Attribution 4.0 International license.

### (iv) Heterologous IgM reactivity.

Heterologous IgM reactivity among the three microbes was observed in 25 samples of this entire study. To identify the original immunoactivity, we employed a multiplex IgG avidity SIA (see below) for the simultaneous determination of infection stages of the three pathogens. In addition, two samples showing SIA IgG and IgM responses against B19 VP2 but not VP1u were resolved by IgM and VP2 ETS EIAs.

### (v) IgG avidity SIAs (singleplex/multiplex).

**FIG 4:**
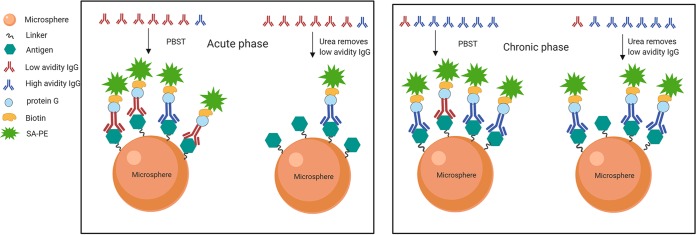
IgG avidity SIA format. The IgG avidity SIA is a chaotrope-based assay for the distinction of the respective primary infections from long-term B-cell immunity. After the sample is incubated with antigens, the immunocomplexes are treated in parallel with or without a protein denaturant. As a result, low-avidity antibodies are separated and eluted away by a wash step, while high-avidity antibodies resistant to urea are retained and finally measured.

The IgG avidity SIAs are based on the principle of elution of the antigen-bound antibodies with urea (Fig. 4), under the experimental conditions presented in Table 8. Briefly, from each serum sample, two dilution series were made in PBST, series 1 (1:20, 1:80, 1:320, and 1:1,280) and series 2 (1:80, 1:320, 1:1,280, and 1:5,120). These dilutions were placed into a 96-well plate and incubated with 1.75 × 10^3^ antigen-coated microspheres/well/analyte for 45 min. Next, series 1 samples were washed three times for 5 min each with 6 M freshly prepared urea (Promega, USA) in PBS, as opposed to series 2, with PBS only. Subsequently, biotinylated protein G (Thermo Scientific, USA) and SA-PE were added, and the MFIs were measured, as for the IgG SIAs (18).

### (vi) Calculation of IgG avidity values.

The IgG avidity values here are the ratios of endpoint titers of series 1 (urea treated) over those of series 2 (non-urea treated), calculated by the curve-fitting software Avidity 1.2 (41).

### (vii) Cutoff determination.

The IgM SIA cutoffs were set at the mean MFIs plus 2 to 5 standard deviations (SDs) of negative controls. For B19, the cutoff was determined with 86 serum samples lacking specific antibodies according to Biotrin’s B19 IgG and IgM EIAs (18, 39). For HCMV and T. gondii, the cutoffs were defined with separate sets of 60 serum samples shown to lack the respective antibodies by the corresponding Architect IgG and IgM tests (18). The IgM cutoff criteria and values are presented in Table 7.

The cutoff values for low and high avidities of IgG for B19, HCMV, and T. gondii are presented in Table 8. For B19 and HCMV, the primary-infection samples were taken within 28 to 50 days after the onset of symptoms, and for T. gondii, samples were taken within 3 months after seroconversion. As defined previously (19), an increase in IgG avidity values (percent units) of ≥10, and simultaneously 2-fold or more, in paired samples (collected within 200 days) was considered significant.

### (viii) Reproducibility.

The intra-assay variability for the IgM SIA was calculated with 8 replicates in the same run, and interassay variability was calculated with 6 distinct runs, employing serum pools containing or lacking the respective IgM. Interassay variability for the IgG avidity SIA was evaluated with 6 to 10 distinct runs during 3 months, using pools containing acute-phase or past-infection serum samples.

### (ix) Statistical analysis.

The positive percent agreement, negative percent agreement, and kappa values between SIAs and the reference assays were calculated with serum panel 1. For the statistical calculations, borderline values in IgM SIAs were considered positive, given the primary role of IgM assays in screening. In IgG avidity SIAs, in turn, borderline-avidity values were considered high-avidity values, due to the important role of these assays in ruling out recent primary infections (30). All the analyses were calculated by 2-by-2 contingency table analysis in GraphPad Prism (GraphPad Software, USA). The overall agreements between SIAs and EIAs were evaluated by kappa values and defined as poor (kappa value of <0.20), fair (0.21 to 0.40), moderate (0.41 to 0.60), good (0.61 to 0.80), and very good (0.81 to 1.00) (44).
