# Supplementary material for: Microsphere-Based IgM and IgG Avidity Assays for Human Parvovirus B19, Human Cytomegalovirus, and Toxoplasma gondii
Source: mSphere. 2020 Mar 18;5(2):e00905-19. doi: 10.1128/mSphere.00905-19 (PMC7082144; doi:10.1128/mSphere.00905-19)
Supplement: TABLE S1 [file mSphere.00905-19-st001.docx]

| **Manufacturer**  Assay | Antigen | Tests | Unit | Interpretation of results |
| --- | --- | --- | --- | --- |
| **Abbott**  Architect CMV IgM | Viral lysate and recombinant antigens | CMIA, two steps indirect anti-IgM detection | Index | Nonreactive, <0.85  Reactive, ≥1  Equivocal, 0.85-0.99 |
| **Abbott**  Architect CMV IgG-avidity | Viral lysate | CMIA, two assays with and without liquid CMV antigen to neutralize high-avidity CMV antibodies | % Avidity | Low avidity, <50%  High avidity, ≥60%  Equivocal, 50-59.9% |
| **BioMérieux**  Vidas CMV IgG-avidity | Viral lysate | ELFA, two assays with and without 6M urea to dissociate low-avidity antibodies | Avidity index | Low avidity, <0.4;  high avidity, ≥0.65;  equivocal, 0.4–<0.65 |
| **BioMérieux**  Vidas Toxo IgM | Cell-cultured tachyzoites lysate | ELFA, two step ELFA with a final fluorescent detection | Index | Negative, <0.55  Positive, ≥0.65  Equivocal, 0.55-<0.65 |
| **BioMérieux**  Vidas Toxo IgG-avidity | Cell-cultured tachyzoites lysate | ELFA, two assays with and without 6M urea to dissociate low-avidity antibodies | Avidity index | Low avidity, <0.2  High avidity, >0.3  Equivocal, 0.2-0.3 |
| **DiaSorin**  LIAISON Biotrin B19 IgM | Parvovirus B19 recombinant VP2 virus-like particle | EIA | Index | Negative <0.9  Positive, >1.1  Equivocal, 0.9-1.1 |
| **In-house**  B19 VP2-IgM | Parvovirus B19 recombinant VP2 virus-like particle | EIA | Absorbance | Negative, <0.17  Positive, >0.22  Equivocal, 0.17-0.22 |
| **In-house**  B19 VP2-IgG-ETS | Parvovirus B19 VP2 virus-like particles and synthetic peptide KYVTGIN | EIA | ETS Index | Recent infection, ≤10%  Past infection, >20%  Equivocal, 11-20 |
| **In-house**  B19 VP1u-IgG-avidity | Parvovirus B19 recombinant protein containing the B19 VP1 unique region | EIA | % Avidity | Low avidity, ≤15%  High avidity, >25%  Equivocal, >15-25 |

CMIA, chemiluminescent microparticle immunoassay; ELFA, enzyme-linked fluorescent assay; EIA, enzyme immunoassay; ETS, epitope type specificity
