# Supplementary figures and images for: Microsphere-Based IgM and IgG Avidity Assays for Human Parvovirus B19, Human Cytomegalovirus, and Toxoplasma gondii
Source: mSphere. 2020 Mar 18;5(2):e00905-19. doi: 10.1128/mSphere.00905-19 (PMC7082144; doi:10.1128/mSphere.00905-19)

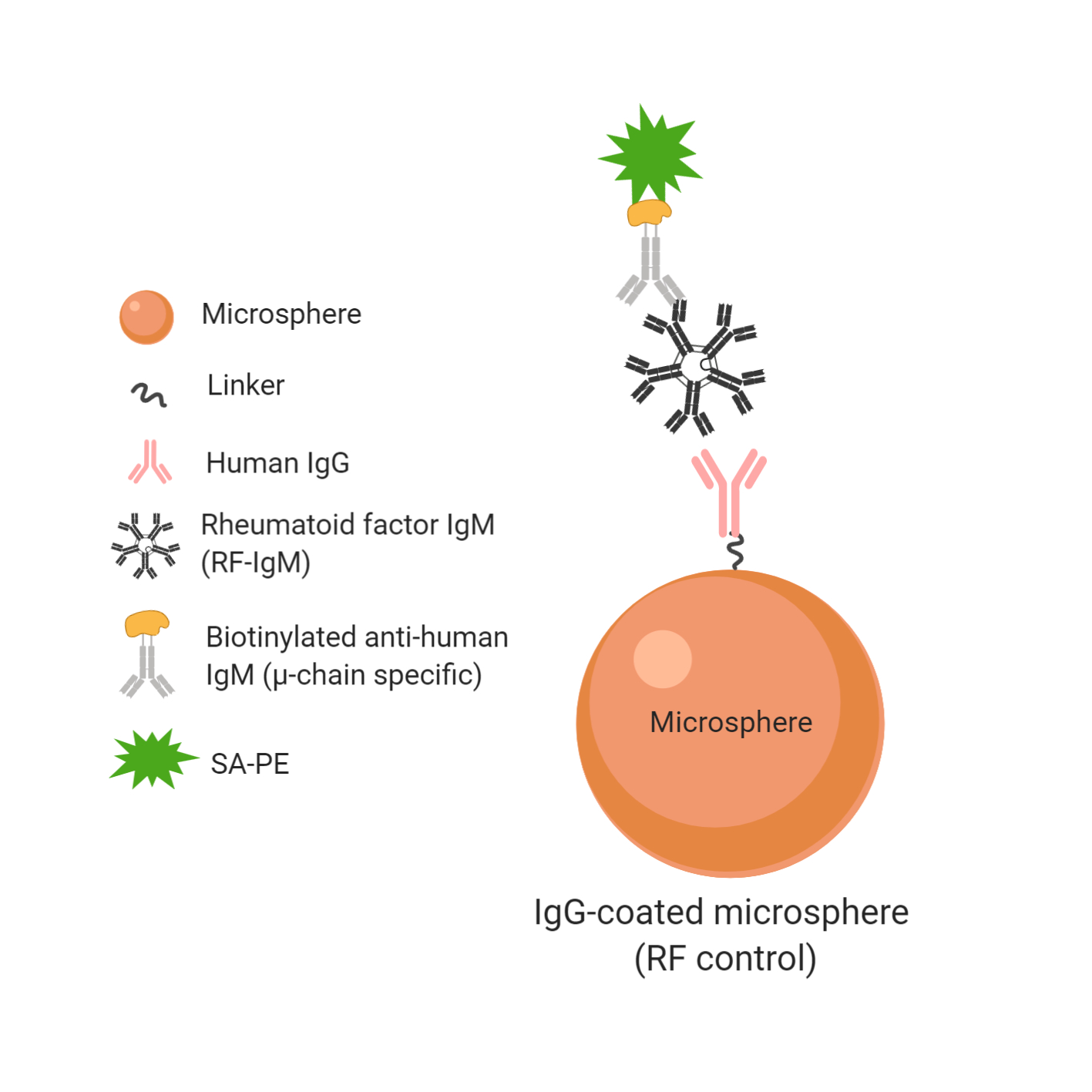

Supplement: FIG S1 [file mSphere.00905-19-sf001.docx]
