# Supplementary material for: Microsphere-Based IgM and IgG Avidity Assays for Human Parvovirus B19, Human Cytomegalovirus, and Toxoplasma gondii
Source: mSphere. 2020 Mar 18;5(2):e00905-19. doi: 10.1128/mSphere.00905-19 (PMC7082144; doi:10.1128/mSphere.00905-19)
Supplement: TEXT S1 [file mSphere.00905-19-s0001.docx]

To study IgG depletion in IgM-SIA, pools containing or lacking specific HCMV-IgM were used. In brief, pools without IgG depletion were diluted in PBST from 1:20 to 1:320 with 4-fold dilution; and pools with IgG depletion were pretreated with GullSORB (yield serum dilution 1:20) and then further diluted in PBST to achieve serum dilutions 1:80 and 1:320. Next, 50µl of diluted sera from each pool were incubated with 1.75x10^3^ HCMV-coated microspheres at room temperature for 45mins. After washes, 50 μl of biotinylated anti-human IgM (Sigma, USA) at 3 μg/ml was added for 30 min. After washes, 50μl of 6 μg/ml SA-PE (Life Technologies, USA) in PBST was applied for 20 min. After final washes, each well was resuspended in 120 μl of PBST and read on a Bio-Plex®200 instrument (Bio-Rad). The MFI values were measured.
