# Supplementary material for: Microsphere-Based IgM and IgG Avidity Assays for Human Parvovirus B19, Human Cytomegalovirus, and Toxoplasma gondii
Source: mSphere. 2020 Mar 18;5(2):e00905-19. doi: 10.1128/mSphere.00905-19 (PMC7082144; doi:10.1128/mSphere.00905-19)
Supplement: Supplementary file 4 [file mSphere.00905-19-sf002.docx]

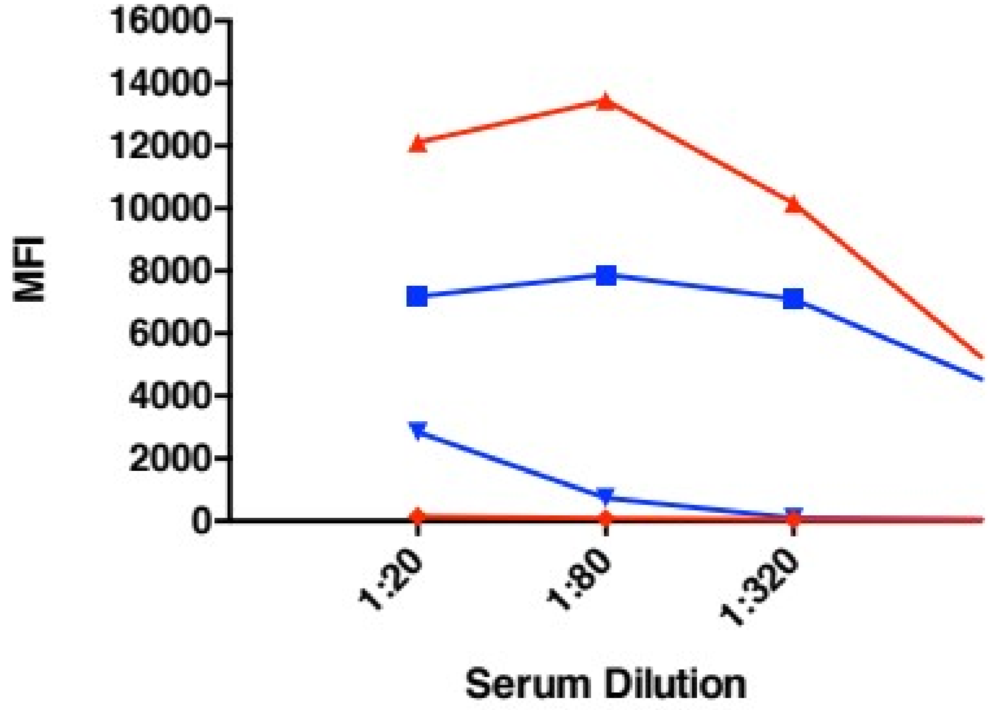

16000
14000
12000
10000
8000
6000
4000
2000
0
MFI
1:20
1:80
1:320
Serum Dilution
